# MicroRNA-28 potentially regulates the photoreceptor lineage commitment of Müller glia-derived progenitors

**DOI:** 10.1038/s41598-017-11112-4

**Published:** 2017-09-12

**Authors:** Hong-Pei Ji, Yu Xiong, Wei-Tao Song, En-Dong Zhang, Zhao-Lin Gao, Fei Yao, Tao Su, Rong-Rong Zhou, Xiao-Bo Xia

**Affiliations:** 1 Department of Ophthalmology, Xiangya Hospital, Central South University, Changsha, 410008 China; 2 Departemnt of Ophthalmology, The People’s Hospital of Guizhou Province, Guiyang, 550002 China; 3 Department of Institute of Medical Sciences, Xiangya Hospital, Central South University, Changsha, 410008 China; 4 Department of Oncology, Xiangya Hospital, Central South University, Changsha, 410008 China

## Abstract

Retinal degenerative diseases ultimately result into irreversible photoreceptor death or loss. At present, the most promising treatment for these diseases is cell replacement therapy. Müller glia are the major glia in the retina, displaying cardinal features of retinal progenitor cells, and can be candidate of seed cells for retinal degenerative diseases. Here, mouse retinal Müller glia dissociated and cultured *in vitro* amplified and were dedifferentiated into Müller glia-derived progenitors (MGDPs), demonstrating expression of stem/progenitor cell markers Nestin, Sox2 and self-renewal capacity. MicroRNAs (miRNAs) play unique roles in the retinogenesis, so we hypothesized miRNAs would contribute to photoreceptor lineage commitment of MGDPs. By TargetScan, Miranda, and Pictar bioinformatics, gain/loss-of-function models, dual luciferase assay, we identified and validated that miR-28 targeted the photoreceptor-specific CRX transcription factor. Anti-miR-28 could induce MGDPs to differentiate into neurons strongly expressing CRX and Rhodopsin, while miR-28 mimic suppressed CRX and Rhodopsin expression. Knockdown of CRX by siRNA blocked the expression of CRX and Rhodospin upregulated by anti-miR-28, indicating that anti-miR-28 potentially induced photoreceptor commitment of MGDPs by targeting CRX, but more experiments are necessary to confirm their role in differentiation.

## Introduction

Retinal degenerative diseases such as age-related macular degeneration (AMD) and retinitis pigmentosa (RP), despite very different etiologies, result into progressive visual impairment associated with photoreceptor damage or loss. These photoreceptors are terminally differentiated neurons and cannot regenerate. For therapy of photoreceptor degenerative diseases, various neuroprotective strategies could be beneficial in the early stages when most photoreceptors are still functional. However, photoreceptor transplantation may be the only effective therapy in the advanced stages when most photoreceptors are irreparably damaged or lost^1^. Photoreceptor transplantation probably is among the most feasible types of neural stem cell (NSC) repair because photoreceptors make short, single synaptic connections to inner retinal circuitry in contrast to most central neurons during integration into complex function circuitry which is a major impediment to replacement of lost neurons^2^.

During neurogenesis, radial glia constitute the majority of multipotent progenitors^3, 4^. In the retina, Müller glia are the major radial cells^5, 6^ and display cardinal features of retinal progenitor cells. In fish, Müller glia can almost regenerate all types of damaged retinal neurons^7–10^. Unfortunately, this regenerative capacity has dwindled during vertebrate evolution to the point that a very limited number of Müller glia can re-enter the cell cycle in rodent retina^11–13^. However, Müller glia possess several advantages such as notably low risk of rejection, fewer ethical problems as therapeutic stem cells compared to embryonic stem cells. Therefore, methods to dedifferentiate Müller glia into MGDPs and direct them to the photoreceptor lineage may facilitate its use as seed cells for photoreceptor regeneration.

MiRNAs are short (21–24 nucleotides) noncoding RNAs that act as crucial regulators of gene transcription by binding to mRNA sequences with partial complementation and inhibiting subsequent translation. MiRNAs are thought to be ideal gene regulators to guide differentiation towards particular cell types due to their ease of transfection, resistance to nuclease action, and long half-life/bioactivity^14^. It is reported that miRNAs play unique roles in the fine-tuning of the proliferation/differentiation of retinal progenitor cells (RPCs) and contribute to the tightly controlled spatial and temporal sequences in retinal development^15^. Therefore, we hypothesized miRNAs might contribute to the differentiation of MGDPs into photoreceptors by binding the 3′ untranslated region (3′ UTR) of the cone-rod homeobox (CRX) gene, which encodes an important transcriptional factor involved in photoreceptor development and maturation^16, 17^. By computer bioinformatics, gain/loss-of-function models and luciferase reporter assays, one identified miRNA, miR-28, was validated to target CRX. Furthermore, transfection of miR-28 inhibitor via lentivirus into MGDPs potentially facilitated commitment to the photoreceptor lineage as evidenced by increased expression of Rhodopsin and CRX. Knockdown of CRX by siRNA blocked the expression of CRX and Rhodospin upregulated by anti-miR-28, indicating that anti-miR-28 induced photoreceptor commitment of MGDPs by targeting CRX. Our results maybe suggest a new view of facilitating Müller glia as seed cells for photoreceptor regeneration in retinal degenerative diseases.

## Results

### Retinal progenitors were derived from mouse Müller glia

**Figure 1:**
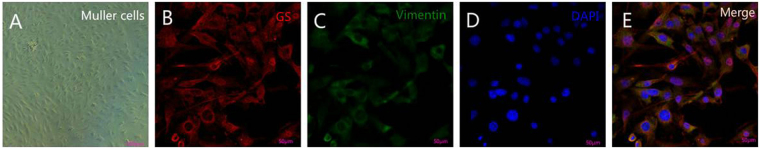
Primary Müller cells cultured *in vitro* expressed the mature phenotype markers GS and Vimentin. (**A**) Passage 2 Müller cells had uniform size and shape, with abundant cytoplasm and well-defined membranes. *Scale bar*: 100 μm. (**B**–**E**) More than 90% cells expresses GS (red) and Vimentin (green) counterstained with DAPI(4′,6-diamidino-2-phenylindole) to highlight nuclei (blue). *Scale bar*: 50 μm.

**Figure 2:**
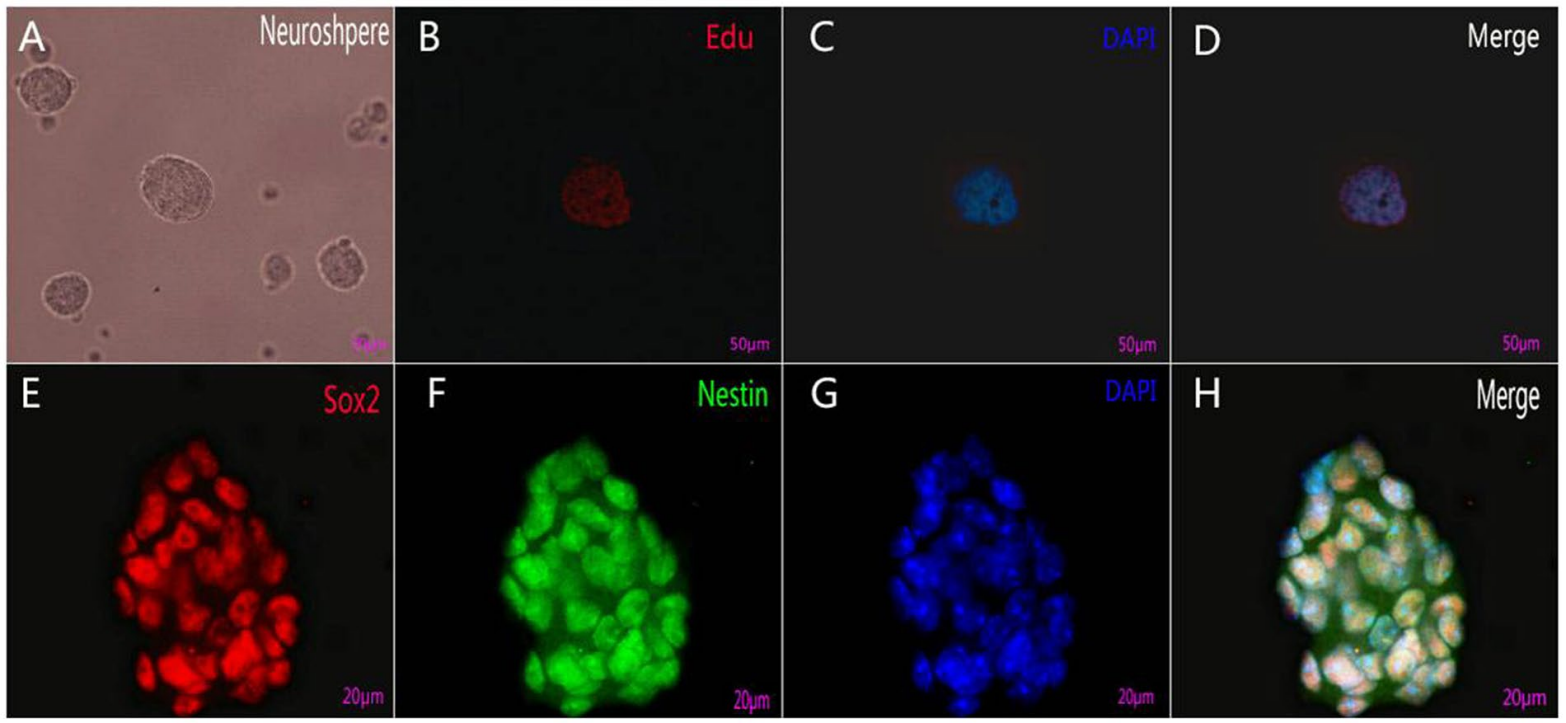
Müller glia cultured under dedifferentiation conditions formed neurospheres containing proliferating cells expressing stem cell markers. (**A**) Neurospheres with good refraction and well defined boundaries. (**B**–**D**) Edu assay showing that many cells had proliferative capacity. (**E**–**H**) MGDPs expressed stem/progenitor cell markers Sox2(red), and Nestin(green) counterstained with DAPI to highlight all neuclei(blue). *Scale bar*: 50 μm (**A**–**D**), 20 μm (**E**–**H**).

We isolated Müller glia from postnatal 5–7 day mice because there is a marked decrease in the proportion of cells re-entering the cell cycle in response to dedifferentiation culture conditions during the second postnatal week^18, 19^. Passage 2 Müller glia cultured *in vitro* had uniform size and shape (Fig. 1A), and more than 90% were immunoreactive for the mature Müller cell markers vimentin and glutamine synthetase (GS) (Fig. 1B–E). Passage 2 Müller glia cultured in dedifferentiation medium for 3–7 days formed neurospheres with good refraction and well-defined boundaries (Fig. 2A). These neurospheres could be passaged more than 3 passages. 5-Ethynyl-2′-deoxyuridine (Edu) assay showed that many cells in passage 2 neurospheres had proliferative capacity (Fig. 2B–D), meanwhile immunocytochemistry demonstrated strong expression of the stem/progenitor cell markers Nestin and SRY (sex determining region Y)-box 2 (Sox2) (Fig. 2E–H). These changes were consistent with Müller glia dedifferentiation into MGDPs.

### MiR-28 targeted CRX

**Figure 3:**
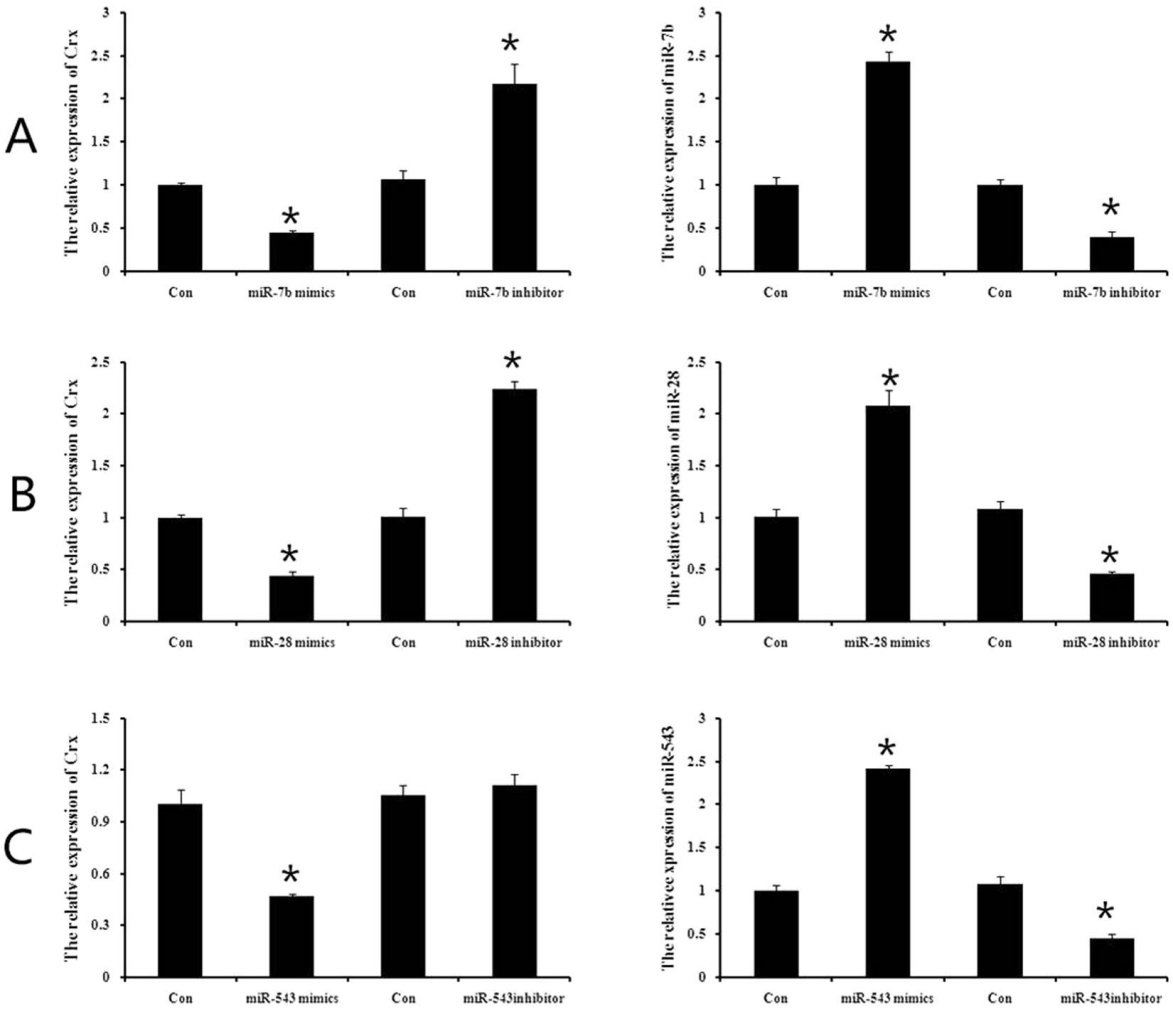
Three of the miRNAs identified by bioinformatics as *CRX*-targeting suppressed CRX expression in MGDPs. CRX **g**ene expression measured by qPCR in cells transfected with miR-7b, miR-543, or miR-28 mimic (left panels) or corresponding inhibitors (right panels). All three mimics suppressed CRX expression whilst inhibition of these miRNAs upregulated CRX expression. None of the other 5 miRNAs identified by bioinformatics modulated CRX expression.

We searched for miRNAs interacting with the 3′UTR of CRX, a transcriptional factor associated with photoreceptor differentiation and maturation, by using Targetscan, Miranda, and Pictar bioinformatics tools. These searches identified 8 miRNAs (miR-7a, miR-7b, miR-28, miR-186, miR-381, miR-876, miR-543, and miR-708) that might target CRX. When respective miRNA mimics were transiently transfected to MGDPs for 2 days, real-time polymerase chain reaction (PCR) demonstrated that only 3 miRNAs, miR-7b, miR-543, and miR-28, suppressed CRX expression (Table 1). When we transfected MGDPs with miR-7b, miR-28, miR-543 mimics and their respective inhibitors (MmiR-AN1287-SN-10, MmiR-AN0622-SN-10, MmiR-AN0362-SN-10, respectively), all three miRNA mimics suppressed CRX expression. Inhibitors for miR-7b and miR-28 upregulated CRX expression as expected (Fig. 3A,B), while miR-543 inhibitor did not upregulate CRX (Fig. 3C) and so was excluded from further analysis.

**Table 1:** The expression of CRX and miRNAs of MGDPs after transfection with miRNAs mimics demonstrated by qPCR assay.

	control	miR-7b mimcs
CRX	1.005 ± 0.09	0.43 ± 0.04^*^
miR-7b	1.00 ± 0.04	2.62 ± 0.16^*^
	Con	miR-186 mimics
CRX	1.05 ± 0.14	1.07 ± 0.09
miR-186	1.00 ± 0.02	1.25 ± 0.02^*^
	Con	miR-7a mimics
CRX	1.02 ± 0.08	0.91 ± 0.016
miR-7a	1.01 ± 0.15	1.15 ± 0.06
	Con	miR-876 mimics
CRX	1.00 ± 0.04	1.14 ± 0.05
miR-876	1.00 ± 0.09	1.25 ± 0.14
	Con	miR-708 mimics
CRX	1.00 ± 0.11	1.02 ± 0.10
miR-708	1.00 ± 0.03	0.89 ± 0.07
	Con	miR-381 mimics
CRX	1.01 ± 0.10	1.25 ± 0.15
miR-381	1.00 ± 0.01	0.83 ± 0.09
	Con	miR-543 mimics
CRX	1.00 ± 0.06	0.39 ± 0.02^*^
miR-543	1.09 ± 0.14	2.56 ± 0.18^*^
	Con	miR-28 mimics
CRX	1.00 ± 0.04	0.36 ± 0.02^*^
miR-28	1.00 ± 0.09	2.08 ± 0.10^*^

*P < 0.05.

**Figure 4:**
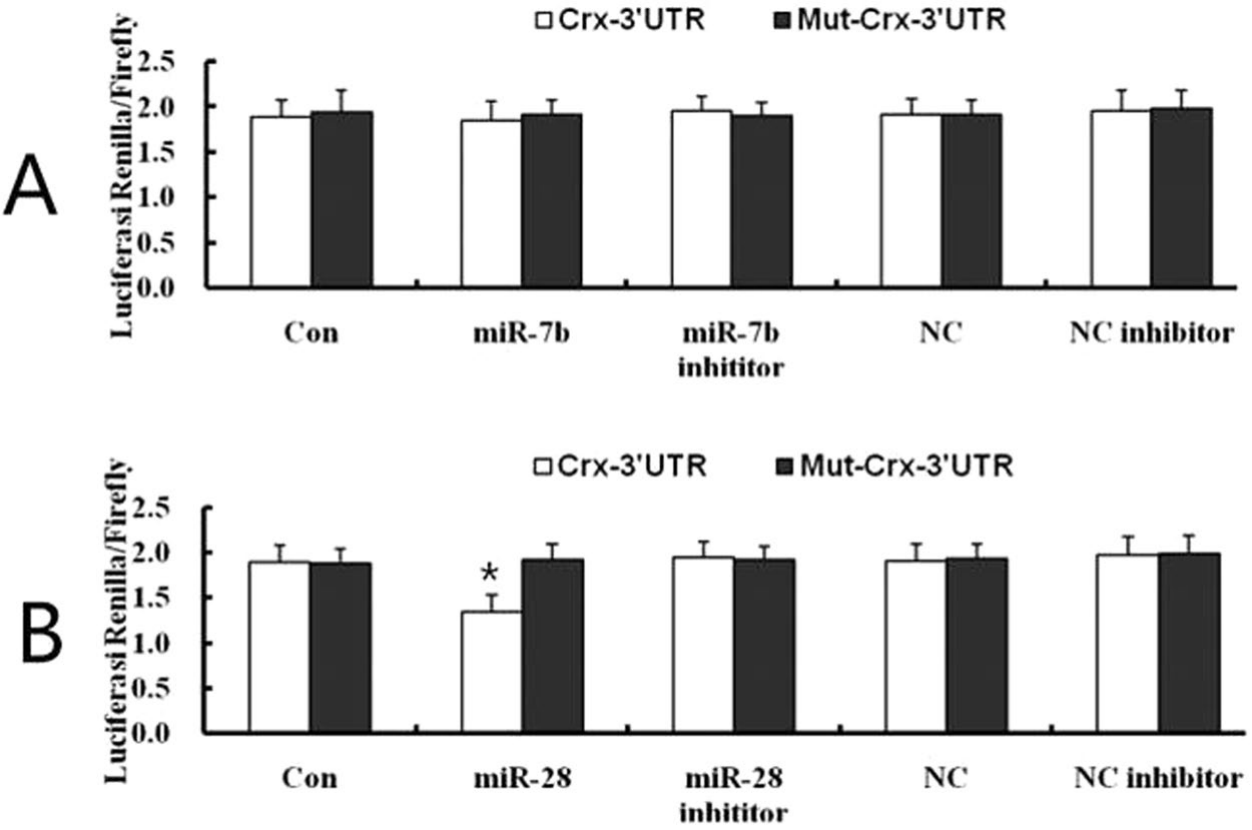
Dual–luciferase reporter assays domenstrated that CRX was target by miR-28. (**A**) There was no obvious change in luciferase activity in cells co-transfected with miR-7b mimic and dual-luciferase reporter plasmid. (**B**) Co-transfection of miR-28 mimic oligonucleotide and the dual-luciferase reporter plasmid containing wt binding sites reduced luciferase activity, while cells co-transfected with miR-28 mimic and reporter plasmid containing mutated binding sites exhibited no change in luciferase activity compared to controls.

To verify whether the 3′UTR of CRX was recognized by miR-28 or miR-7b, we generated luciferase reporter constructs containing a CRX 3′UTR with either wild type (wt) or mutated type of miR-28 (or miR-7b) binding sites. As shown in Fig. 4, there was no obvious change in luciferase activity in cells co-transfected with miR-7b mimic and dual-luciferase reporter plasmid (Fig. 4A). Co-transfection of miR-28 mimic and the dual-luciferase reporter plasmid containing wt binding sites reduced luciferase activity, while cells co-transfected with miR-28 mimic and reporter plasmid containing mutated binding sites exhibited no change in luciferase activity compared to controls (Fig. 4B). These results indicated that the putative binding site seed sequences of CRX were specifically recognized by miR-28, but not by miR-7b. Therefore, we selected miR-28 for further study on MGDP differentiation.

### miR-28 potentially induced photoreceptor commitment of mice MGDPs

**Figure 5:**
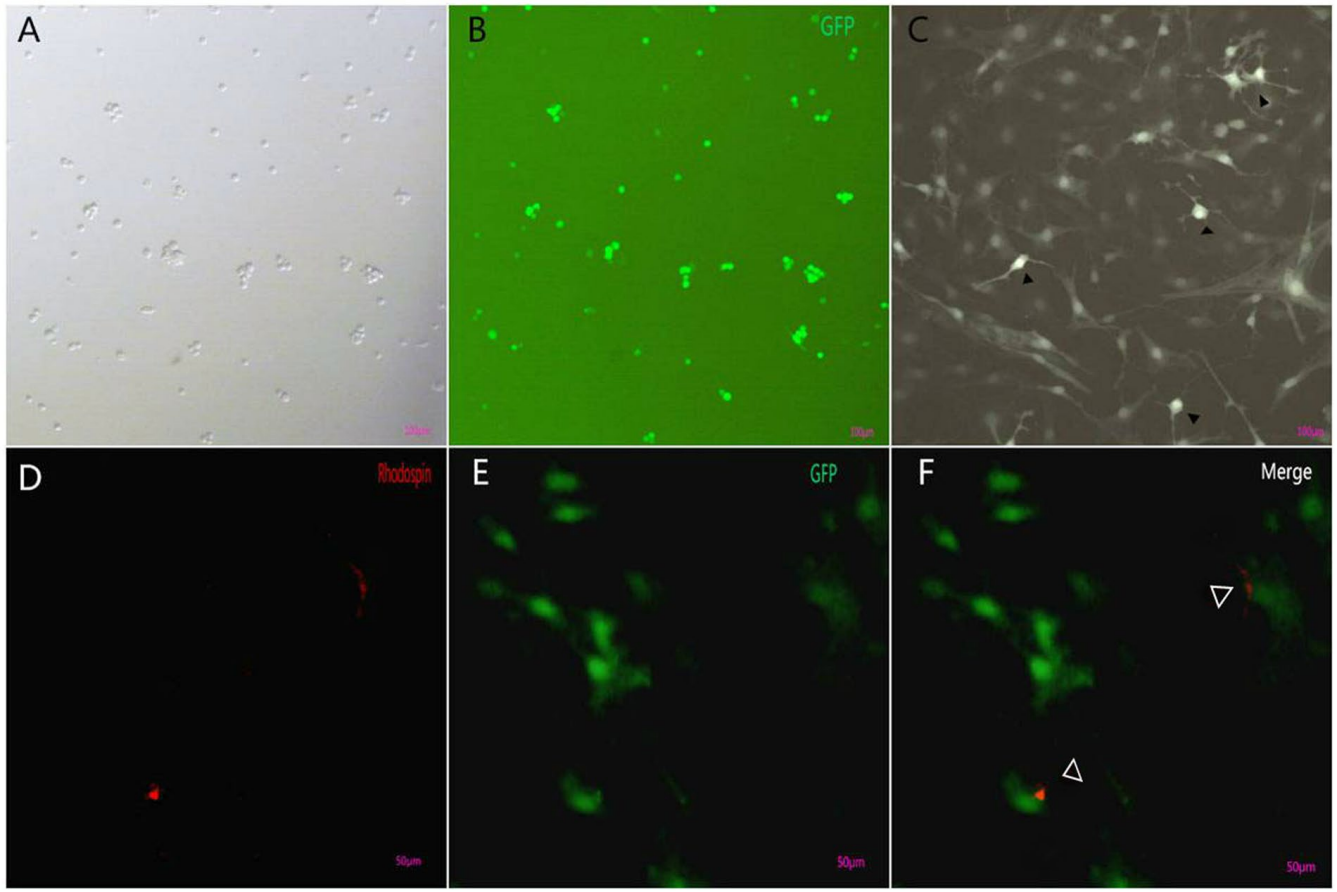
Differentiation of MGDPs into neuron-like cells. (**A**,**B**) After 60 h transfection with the lentivirus mU6-MCS-Ubi-anti-miR-28-EGFP, more than 80% of MGDPs were successfully infected and expressed enhanced green fluorescent protein (EGFP, green). (**C**) MGDPs infected with mU6-MCS-Ubi-anti-miR-28-EGFP encoding the miR-28 inhibitor differentiated into cells with neuron-like features, including condensed cytoplasm, one or more synaptic processes, and a reduced cytoplasm:nucleus ratio (black triangles). (**D**–**H**) Some MGDPs differentiated into photoreceptors expressing Rhodopsin (red triangle). *Scale bar*: 100 μm (**A**–**C**), 50 μm (**D**–**F**).

**Figure 6:**
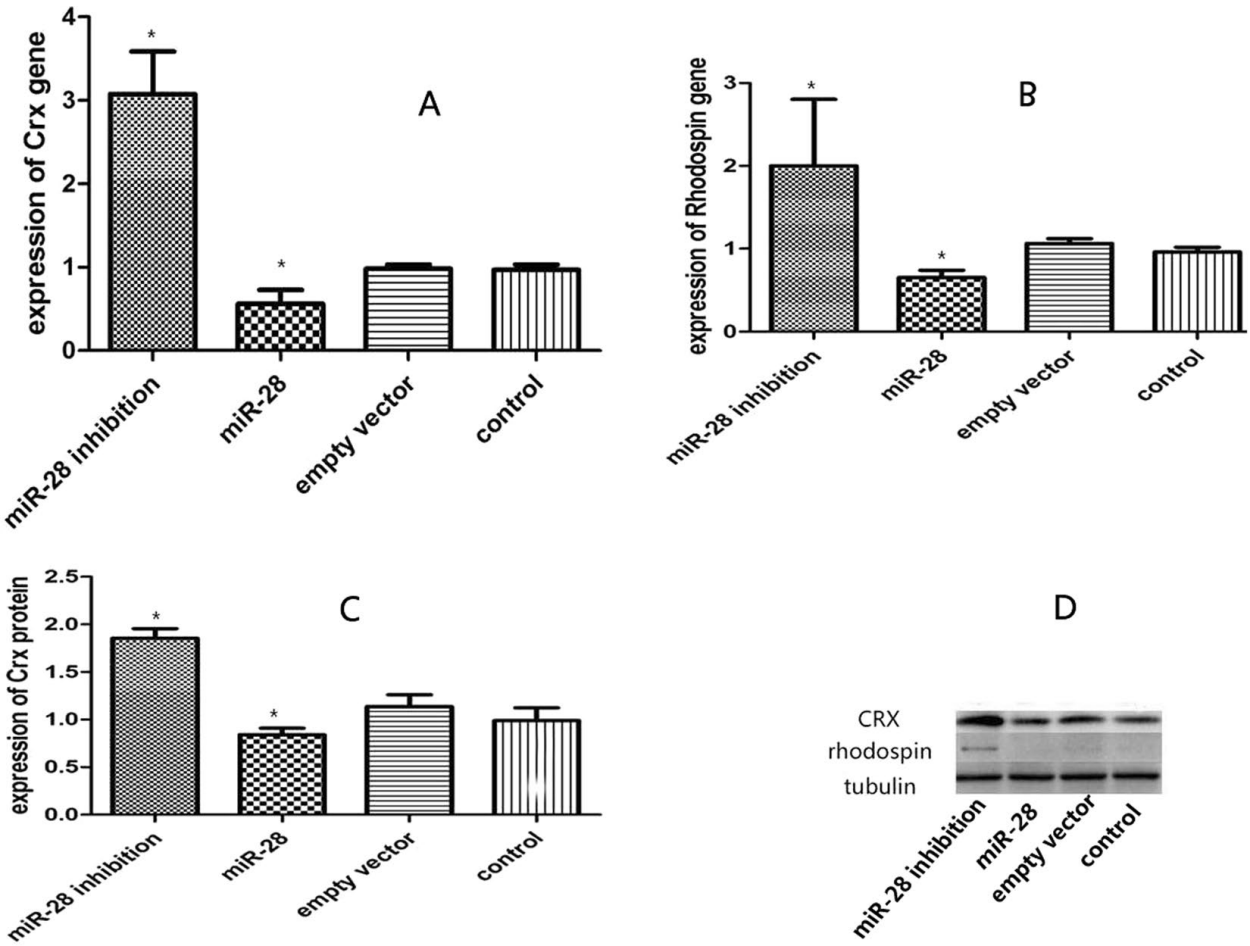
MicroRNA-28 regulates MGDP differentiation into rods. (**A**) Quantitative PCR analysis showing upregulation of CRX by the miR-28 inhibitor and CRX downregulation by the miR-28 mimic in neurospheres. (**B**) Quantitative PCR showing upregulation of Rhodopsin by the miR-28 inhibitor and downregulation by the miR-28 mimic. (**C**,**D**) Sample western blot and densitometric analysis demonstrating upregulation of CRX and Rhodopsin proteins by the miR-28 inhibitor and downregulation by the miR-28 mimic.

To study the effects of miR-28-mediated CRX modulation on differentiation of MGDPs, MGDPs isolated from passage 2 neurospheres were infected with lentivirus containing miR-28 mimic or inhibitor. After 60 h, more than 80% of MGDPs were successfully infected as indicated by EGFP expression (Fig. 5A,B). For 7 days of differentiation, some MGDPs transfected with miR-28 inhibitor exhibited neuronal features, including condensed cytoplasm, one or more synaptic processes, and reduced cytoplasm:nucleus ratio (Fig. 5C), consistent with Jayaram H^20^. Furthermore, some cells demonstrated expression of the special photoreceptor marker Rhodopsin. Expression levels of mRNAs encoding the photoreceptor markers CRX (Fig. 6A) and Rhodopsin (Fig. 6B) were enhanced by miR-28 inhibition and suppressed by miR-28 mimic as assessed by real-time PCR. These mRNA expression changes were paralleled by protein expression changes measured by western blot (Fig. 6C,D). In contrast to Rhodopsin, these neuron-like cells did not express S-opsin by immunocytochemistry assay, and S-opsin protein was also not detected by western blot assay (not shown).

To investigate whether anti-miR-28 induced differentiation of MGDPs toward photoreceptor directly through CRX, MGDPs transfected with lenivirus mU6-MCS-Ubi-anti-miR-28-EGFP for 3 days were again transfected with siRNA harbouring no 3′UTR of CRX. Western blot demonstrated that si-CRX specifically knocked down the expression of CRX. The expression of CRX protein upregulated by anti-miR-28 was also partly blocked by CRX siRNA (Fig. S1) and Rhodopsin protein was not detected (data was not shown), indicating that anti-miR-28 could have a potential to drive MGDPs to photoreceptor differentiation by targeting CRX, but more experiments are necessary to confirm its role in differentiation.

## Discussion

We demonstrated that anti-miR-28 potentially induced photoreceptor lineage commitment of MGDPs as evidenced by neuron-like morphological changes and upregulation of the photoreceptor markers CRX and rhodopsin at both the mRNA and protein levels. The expression of CRX and Rhodopsin protein were blocked by CRX siRNA, suggesting anti-miR-28 induced MGDPs to differentiate into photoreceptors commitment by targeting CRX. The CRX gene is the earliest known photoreceptor markers, regulating development and maturation of rods and cones. Besides postnatal rod, the embryonic-stage CRX-positive donor cells can also integrate within the outer nuclear layer of retina and differentiate into new rods and cones, which are critical to visual function. The ratio of rods to cones is similar to that of the host retina (20:1)^21, 22^. In this study, we did not detect the expression of S-opsin, the marker of cones, in contrast to rhodospin. Maybe too few cones were generated for finding. Alternatively, it is unlikely that our methodology was inappropriate as we tested primers from 3 different companies (unpublished observations).

Although orthodenticle homeobox 2 (OTX2) rather than CRX is the key regulatory factor of photoreceptor fate, overexpression of CRX is also capable to induce the retinal progenitors and retinal endogenous stem cells such as the iris or ciliary-derived cells to differentiate to light-sensitive photoreceptor phenotypes *in vivo* and vitro. While cornea-derived stem cells by misexpression of CRX only express photoreceptor-specific protein not to be functional, and none of neural stem cells from hippocampus acquires Rhodopsin immunoreactivity^23–28^. Retinal Müller glia are also knows as one of endogenous stem cells and could be induced to photoreceptor phenotypes by upregulation of CRX regulated by anti-miR-28 as demonstrated in our study. Our results further proved that retinal Müller glia has capacity to facilitate photoreceptor regeneration, consistent with the literatures^5, 20^, but we did not go a step further to detect photoreceptor function, which was one of limits of our study, and more experiments are needed to confirm the role of anti-miR-28 in differentiation.

In the retinogenesis, miRNAs play unique roles in the fine-tuning of the proliferation/differentiation of RPCs and contribute to the tightly controlled spatial and temporal sequences in development^15^. Sole miRNA even can induce the commitment of neuronal differentiation of stem/progenitor cells, for example, anti-miR-410 can induce retinal pigment epithelium differentiation in stem cells^29, 30^. Ectopic miR-124 expression is sufficient to induce MGDPs differentiate to early neuronal lineages^31^. We similarly demonstrated that anti-miR-28 potentially induced photoreceptor lineage commitment of MGDPs, which further proved the viewpoint that distinct miRNAs must be down-regulated to generate the latest neuron types^32^.

In conclusion, anti-miR-28 potentially induce MGDPs to differentiate to photoreceptor commitment by targeting CRX, suggesting a new view of facilitating Müller glia as seed cells for photoreceptor regeneration in retinal degenerative diseases. But more study was needed to further assess cone differentiation of MGDPs and whether these differentiated photoreceptor-like cells can integrate in the host retina and develop mature.

## Methods

### Animals (Ethics statement)

All animal experiments in this study conformed to the Guidelines for Animal Experiments of Central South University, Changsha, China, and were conducted with the approval of the Animal Research Committee, Xiangya School of Medicine, Central South University (Permit No. SCXK 2006–0002).

### Cell Culture

Müller glia were isolated and dedifferentiated according to previous methods^33–35^. Briefly, C57BL/6J mice (5–7 days old) were sacrificed by cervical dislocation under isoflurane anesthesia. Eyes were enucleated and neuroretina dissected without disrupting the underlying retinal pigmented epithelium starting 0.5 mm from the ora serrata to avoid contamination by retinal stem cells at the ciliary margin. Neuroretinas were dissociated into small aggregates and digested by trypsin-EDTA (0.25% trypsin, 2% EDTA, Gibco, USA) for 5 minutes. Digestion was stopped by addition of Dulbecco’s modified Eagle’s medium F12 (DMEM/F12, HyClone, USA) supplemented with 15% fetal bovine serum (FBS, Gibco). Cell suspensions were filtrated through a 0.75 μm stainless steel sieve and centrifuged at 1000 rpm for 5 min. The cell pellets were resuspended in DMEM/F-12 containing 15% FBS, seeded on 25 cm^2^ flasks, and grown at 37 °C under a 5% CO_2_ atmosphere. The cells were passaged via trypsinization every 4–5 days. Passage 2 Müller cells were used in the experiments.

The neurosphere assay is frequently used to isolate and propagate neural stem cells. We generated neurospheres by standard techniques. Passage 2 Müller cells were dissociated using Accutase (Invitrogen, USA) and cultured on ultra low attachment 6-well plates(Corning, USA) in a serum-free dedifferentiation medium containing DMEM/F12 (Gibco), 1 × N2 supplement (Gibco), 2 × B27 supplement without Vitamin A (Gibco), 20 ng/ml murine epidermal growth factor (EGF, Peprotech, USA), 10 ng/ml murine basic fibroblast growth factor (bFGF2, Peprotech), and 2 mM L-glutamine (HyClone) at 1 × 10^5^ cells/cm^2^. Half of the dedifferentiation medium was changed every other day.

### Immunocytochemical analysis

Immunocytochemical analysis was performed as previously described. Briefly, the cultured cells or neurospheres were fixed in 4% paraformaldehyde-PBS for 30 min, blocked in PBS containing 5% goat serum and 0.3% TritonX-100 at 37 °C for 1 h, and then incubated with one of the following primary antibodies overnight at 4 °C: rabbit anti-GS (1:100, Abcam, ab73593), mouse anti-vimentin (1:75, Abcam, ab8976), rabbit anti-Sox2 (1:100, Abcam, ab92494), mouse anti-nestin (1:100, Abcam, ab6320), mouse anti-rhodopsin (1:50, Abcam, ab5417). Following PBS wash, cells or neurospheres were incubated in fluorophore-conjugated goat anti-mouse IgG (ZSGB-BIO, China) or goat anti-rabbit IgG (Multi Sciences, China) for 2 hours in the dark and then counterstained with 4′,6-diamidino-2-phenylindole (DAPI; Sigma, USA) for five minutes. Fluorescent images were recorded using confocal microscopy (Leica SP8, Germany) or fluorescent microscopy (Leica DM5000B, Germany).

### Edu proliferation assay

For the Edu proliferation assay, neurospheres were seeded on poly-L-lysine-coated slides. After 24 h, the slides were processed according to the manufacture’s instructions (Ribobio, China). Fluorescent images were recorded by confocal or fluorescent microscopy.

### MicroRNA prediction

The bioinformatics tools TargetScan (http://www.targetscan.org), Miranda (http://www.mircrona.org/microrna/home.do), and Pictar (http://pictar.mdc-berlin.de) were used to search for miRNAs that target the CRX 3′ UTR. These algorithms were selected because they provided complementary information. MiRNAs were predicted by these bioinformatics individually, and the overlapping miRNAs were chosen for subsequent experiments. The selected miRNAs mimics or inhibitions (Genecopoeia, USA) were transiently transfected to MGDPs, those that were not negatively related to CRX were excluded from further study.

### Target validation

To validate CRX targeting by miRNAs showing the expected downregulation of CRX in MGDPs, dual-luciferase assays were conducted. MGDPs were transiently transfected with miRNA mimics or inhibitors (Genecopoeia, USA) using lipofectamine 2000 (Invitrogen) and with psiCHECKTM-2 vectors (Auragene, China) encoding wild type or mutant type of miRNA binding sites to the 3′UTR of CRX (NM_001113330.1) upstream of a synthetic firefly luciferase gene. The primers used for generation of seed sequences inserted into psiCHECKTM-2 vectors were as follows: mmu-miR-7b seed sequence, CRX-3′UTR-7b forward primer containing a Xho l restriction site (5′-CCGCTCGAGGCTGGGATGCTGTGGCTTGTA-3′) and reverse primer containing an NotI restriction site (5′-GTTGCGGCCGCCTGGGCTGGTAGTCTTGGGTT-3′); mut-CRX-3′UTR-7b, forward primer containing a NotI restriction site (5′-GTTGCGGCCGCGCTGGGATGCTGTGGCTTGTA-3′) and reverse primer containing a XhoI restriction site (5′-CCGCTCGAGCTGGGCTGGTAGTCTTGGGTT-3′); mmu-miR-28 seed sequence, CRX-3′UTR-28 forward primer containing a Xho l restriction site (5′-CCGCTCGAGTACTGGCTTGCTTCCTCT-3′) and reverse primer containing an Not I restriction site (5′-GTTGCGGCCGCTCTACCTCCCTCGTGTTG-3′); mut-CRX-3′UTR-28, forward primer containing a NotI restriction site (5′-GTTGCGGCCGCTACTGGCTTGCTTCCTCT-3′) and reverse primer containing a XhoI restriction site (5′-CCGCTCGAGTCTACCTCCCTCGTGTTG-3′). Luciferase activity was measured using a Dual-Glo luciferase assay kit (Promega, USA) according to the manufacturer’s instructions. The data were normalized by dividing the firefly luciferase activity by that of the Renilla luciferase activity.

### MicroRNA-28 lentivirus vector construction and transfection of MGDPs

The mouse pre-miR-28 sequence was amplified from genomic DNA of mouse fibroblasts and cloned into the lentivirus expression vector mU6-MCS-Ubi-EGFP (provided by Gene, China). Primers used for amplification were as follows: Mmu-miR-28-5p-inhibitor, forward 5′-AAGGAGCTCACAGTCTATTGAG-3′ and reverse 5′-CTCAATAGACTGTGAGCTCCTT-3′; miR-28, 5′-ATTGAGTTGCCTTTCTGATT CTCCCACTAGATTGTGAGCTGCTGGAGGGCAGGCACT-3′ and reverse 5′-AGTGCCTGCCCTCCAGC AGCTCACAATCTAGTGGGAGAATCAGAAAGGCAACTCAATAGACTGTGAGCTCC TTGAAGGTAGGGACC -3′.

293T cells were transfected with lentiviral plasmid (empty EGFP control, miR-28, or miR-28 inhibitor), as well as lentiviral packaging plasmids including pLP1 and pLP2 using the FuGENE^®^ HD transfection reagent (Roche). Viral -bearing supernatant was collected and cellular debris removed by syringe filtering (0.45 μm pore size; Millipore). To improve the transfection efficiency, passage 2 neurospheres were digested by Accutase into single cell suspensions and cultured as monolayer at 4 × 10^5^ MGDPs per well in 6-well plates (Beaver, China) with serum-free NSC medium. MGDPs were divided into four groups: A (control group with no lentivirus transfection), B (empty vector group transfected with lentivirus mU6-MCS-Ubi-EGFP), C (miR-28-overexpression group transfected with lentivirus mU6-MCS-Ubi-miR-28-EGFP), and D (anti-miR-28 group transfected with lentivirus mU6-MCS-Ubi-anti-miR-28-EGFP). After 24 h, the medium in group B, C, and D cultures were replaced by 600 μl fresh medium with virus at a MOI of 10 and 5 μg/ml polybrene, while group A was replaced with new medium containing 5 μg/ml polybrene, but without virus. After 36 h, all cultures were switched to differentiation medium containing Neurobasal medium plus B27 supplement (with vitamin A, Gibco, USA)). Thereafter, half of the medium was changed every other day for 7 days.

### Samll interfering RNA(siRNA) against CRX and transfection

MGDPs transfected with lentivirus mU6-MCS-Ubi-anti-miR-28-EGFP or mU6-MCS-Ubi-EGFP for 3 days were again transfected with siRNA harbouring no 3′UTR of CRX (5′-GCATCTCAGATTCTTACAG-3′, Genechem, China) or its negative control siRNA (si-NC) using lipofectamine 2000 (Invitrogen, USA) according to the manufacture’s recommendation. 4 days later, the expression of CRX and Rhodopsin protein were examined by western blot assay.

### Quantitative RT-PCR

For mRNA expression analysis, total RNA were isolated using the RNeasy kit (Bioflux, Japan). For miRNA expression analysis, RNA was extracted using the Qiagen RNeasy kit (Qiagen). RNA was reverse transcribed using the Access RT-PCR system (Promega, USA). Quantitative PCR was conducted using the All-in-oneTM qPCR Mix (GeneCopoeia, USA). Quantification of gene expression was based on the 2^−ΔΔCt^ method. Target gene expression levels were normalized to β-actin expression, while miRNA expression levels were normalized to U6 (MmiRQP9002, Genecopoeia, USA) expression. The gene primer sequences for CRX were forward 5′-TCACCAAAGGAAGGCAGAAC-3′ and reverse 5′-GTCAGAGGAAGCCCAGT-3′, the rhodopsin gene sequences were forward 5′-ATGTTCCTGCTCATCGTGCT-3′ and reverse 5′-TGTGTAGAGGGTGGTGGTGA-3′, and the β-actin gene primer sequences were forward 5′-GTGGGGCGCCCCAGGCACCA-3′ and reverse 5′-CTCCTTAATGTCACGCACGATTTC-3′.

### Western blot analysis

Proteins were extracted using the radio-immunoprecipitation assay (RIPA) buffer (RiboBio; China) containing a protease inhibitor cocktail (Sigma, USA). Lysate proteins (50 μg protein per gel lane) were separated by 15% SDS-PAGE and transferred onto polyvinylidene fluoride (PVDF) membranes (280 mA for 55 min). The membranes were blocked with 5% skimmed milk in Tris buffered saline with 0.1% Tween-20 (TBS-T) for 1 h at 37 °C, then incubated with primary antibodies(mouse anti-rhodopsin, 1:500; rabbit anti-CRX, 1:500, Santa Cruz, sc-30150; rabbit anti-S-opin, 1:20, Abcam, ab81017) overnight at 4 °C. Immunoblotting with a mouse α-tubulin antibody (1:10000, Proteintech, 66031-1-Ig) was used as the gel loading control. After several washes, the membranes were incubated with horseradish peroxidase (HRP)-conjugated goat anti-mouse IgG (Multi Science, China) or HRP-goat anti-rabbit IgG (Multi Science, China). The bands were semiquantified by densitometry using Bio-Rad imaging software.

### Statistical analysis

The data are presented as the mean ± SD. Multiple treatment group means were compared by one-way analysis of variance with pair-wise comparisons by LSD-t assay. Paired means were compared by Student’s t-test. In all tests, a P < 0.05 was considered statistically significant. Graphpad Prism 6.0 software was used for all statistical analyses and data plotting.

### Availability of data and materials

The dataset supporting the conclusions of this article is included within the article.

## Electronic supplementary material

supplementary information
