# Supplementary material for: MicroRNA-28 potentially regulates the photoreceptor lineage commitment of Müller glia-derived progenitors
Source: Sci Rep. 2017 Sep 12;7:11374. doi: 10.1038/s41598-017-11112-4 (PMC5595954; doi:10.1038/s41598-017-11112-4)

*MicroRNA-28 regulates the photoreceptor lineage commitment of Müller glia-derived progenitors*

Hong-Pei Ji, Yu Xiong, Wei-Tao Song, En-Dong Zhang, Zhao-Lin Gao, Fei Yao, Tao Su, Rong-Rong Zhou, Xiao-Bo Xia.

**Fig. S1** anti-miR-28 induced differentiation of MGDPs by targeting CRX gene. Western blot demonstrated that si-CRX could be specifically knocked down the expression of CRX. The expression of CRX protein upregulated by anti-miR-28 was also partly blocked by CRX siRNA.


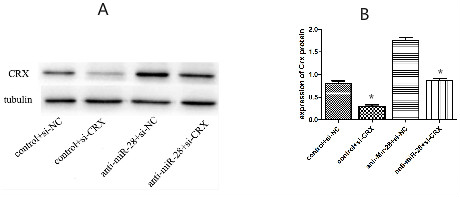

Supplement: Supplementary file 1 — supplementary information [file 41598_2017_11112_MOESM1_ESM.doc]
